# The Adoption of a COVID-19 Contact-Tracing App: Cluster Analysis

**DOI:** 10.2196/41479

**Published:** 2023-06-20

**Authors:** Tessi M Hengst, Lilian Lechner, Laura Nynke van der Laan, Arjen Hommersom, Daan Dohmen, Lotty Hooft, Esther Metting, Wolfgang Ebbers, Catherine A W Bolman

**Affiliations:** 1 Department of Psychology Open University Heerlen Netherlands; 2 Department of Communication and Cognition Tilburg University Tilburg Netherlands; 3 Department of Science Open University Heerlen Netherlands; 4 Department of Management Science Open University Heerlen Netherlands; 5 Department of Epidemiology University Medical Center Utrecht Utrecht Netherlands; 6 Data Science Center in Health University Medical Center University of Groningen Groningen Netherlands; 7 Department of General Practice and Elderly Medicine University Medical Center University of Groningen Groningen Netherlands; 8 Department of Social and Behavioral Sciences Erasmus University Rotterdam Netherlands

**Keywords:** contact-tracing app, CTA, CoronaMelder, intention, adoption, cluster analysis, application, psychosocial, data, risk, societal, social norm, norm, COVID-19, adaptation, acceptance, mHealth, mobile health

## Abstract

**Background:**

During the COVID-19 pandemic, there was limited adoption of contact-tracing apps (CTAs). Adoption was particularly low among vulnerable people (eg, people with a low socioeconomic position or of older age), while this part of the population tends to have lesser access to information and communication technology and is more vulnerable to the COVID-19 virus.

**Objective:**

This study aims to understand the cause of this lagged adoption of CTAs in order to facilitate adoption and find indications to make public health apps more accessible and reduce health disparities.

**Methods:**

Because several psychosocial variables were found to be predictive of CTA adoption, data from the Dutch CTA CoronaMelder (CM) were analyzed using cluster analysis. We examined whether subgroups could be formed based on 6 psychosocial perceptions (ie, trust in the government, beliefs about personal data, social norms, perceived personal and societal benefits, risk perceptions, and self-efficacy) of (non)users concerning CM in order to examine how these clusters differ from each other and what factors are predictive of the intention to use a CTA and the adoption of a CTA. The intention to use and the adoption of CM were examined based on longitudinal data consisting of 2 time frames in October/November 2020 (N=1900) and December 2020 (N=1594). The clusters were described by demographics, intention, and adoption accordingly. Moreover, we examined whether the clusters and the variables that were found to influence the adoption of CTAs, such as health literacy, were predictive of the intention to use and the adoption of the CM app.

**Results:**

The final 5-cluster solution based on the data of wave 1 contained significantly different clusters. In wave 1, respondents in the clusters with positive perceptions (ie, beneficial psychosocial variables for adoption of a CTA) about the CM app were older (*P*<.001), had a higher education level (*P*<.001), and had higher intention (*P*<.001) and adoption (*P*<.001) rates than those in the clusters with negative perceptions. In wave 2, the intention to use and adoption were predicted by the clusters. The intention to use CM in wave 2 was also predicted using the adoption measured in wave 1 (*P*<.001, β=–2.904). Adoption in wave 2 was predicted by age (*P*=.022, exp(B)=1.171), the intention to use in wave 1 (*P*<.001, exp(B)=1.770), and adoption in wave 1 (*P*<.001, exp(B)=0.043).

**Conclusions:**

The 5 clusters, as well as age and previous behavior, were predictive of the intention to use and the adoption of the CM app. Through the distinguishable clusters, insight was gained into the profiles of CM (non)intenders and (non)adopters.

**Trial Registration:**

OSF Registries osf.io/cq742; https://osf.io/cq742

## Introduction

During the COVID-19 pandemic, governments took measures to prevent the spread of the coronavirus. An example of these measures is the development and implementation of several national and international apps. On the one hand, these apps were designed to register vaccination certificates. On the other hand, contact-tracing apps (CTAs) were developed. CTAs can be defined as “software that can be installed on a user’s device, such as a smartphone, to notify the user when he or she comes into contact with a person infected with SARS-CoV-2” [1]. In the Netherlands, the CTA used is called CoronaMelder (CM). The CM app uses Bluetooth to record the people someone is around for more than 15 minutes at a distance of less than 1.5 m [2]. If this user later tests positive and reports this to the Dutch health authority, the Gemeentelijke Gezondheidsdienst (GGD), all persons who were around that user and have CM on their phone will receive a notification in the app, accompanied by instructions on what to do in the event of such notification.

Although the literature shows that lower adoption of a CTA can be effective [3], Trang et al [4] found that more than 50% of the population should install and use a CTA to effectively suppress the transmission of the coronavirus. However, in Europe, the uptake of CTAs ranged between 1% and 50% of the population [5]. In the Netherlands, the Dutch government found that only 28% of the Dutch population adopted (ie, downloaded the technology) the CM app since its launch [6]. Adoption is the lowest among people with a lower education level, with lower monthly incomes, with an immigrant background, and aged over 80 years. These populations tend to have less access to information and communication technology (ie, the digital divide) and are more vulnerable to COVID-19 infection [7,8], which highlights the importance of adoption among these vulnerable populations. Hence, it is important to understand the factors that promote or hinder the adoption of CTAs, such as the CM app, to facilitate adoption and find indications to make public health apps more accessible and reduce health disparities.

Previous research during the COVID-19 crisis has already shown that there are several determinants for the adoption of CTAs [1,5,9-16]. From those studies, 6 psychosocial perceptions have emerged that influence the adoption of CTAs: trust in the government, beliefs about personal data, social norms, perceived personal and societal benefits, risk perceptions, and self-efficacy. Zetterholm et al [8] and Van Der Waal et al [17] found that these determinants of the adoption of CTAs align with the unified theory of acceptance and use of technology (UTAUT) and the Health Belief Model (HBM), which are therefore regularly used to predict and explain the adoption of CTAs [7,11,16].

UTAUT is a technology acceptance model that predicts the (intention of) technology acceptance by factors that enhance or impede the acceptance and use of technology from the viewpoint of the user, categorized into 4 predictors; performance expectancy, effort expectancy, social influence, and facilitating conditions [18,19]. These predictors determine the behavioral intention, which in turn results in usage behavior. However, each of these predictors may be influenced by the gender, age, experience, and voluntariness of the user [19]. The HBM focuses on health behavior and health communication interventions specifically [20]. Beliefs involved are perceived susceptibility, severity, benefits, and barriers. The perceptions of these 4 beliefs can predict one’s behavior, complemented by health motivation and cues to action [20]. However, the beliefs may be influenced by demographic variables and psychological characteristics.

According to Zetterholm et al [8], CTA adoption is predicted by the HBM-related determinants perceived susceptibility, self-efficacy, and perceived benefits. In line with this, van der Waal et al [17] found self-efficacy and perceived benefits to be associated with CTA adoption. In addition, the UTAUT-related variables performance expectancy and social influence (which have similarities with the CTA adoption determinant social norms) were found to relate to CTA adoption [17]. Hence, UTAUT and the HBM confirm several determinants for CTA use posited in the literature.

These studies consider psychosocial perceptions as individual predictors of intention and behavior. Yet, we assume these perceptions together reflect certain profiles that are predictive of the mentioned outcomes. These profiles might also relate to certain demographic characteristics. To examine how these psychosocial perceptions hold up within psychosocial profiles, in this study, we conducted a cluster analysis. This exploratory statistical method is repeatedly used in health psychology to identify groups of people at risk of developing medical conditions and at risk of poor outcomes [21]. In the current context, it can provide an overview of the variety of users of the CM app and the way in which they differ from each other. Hence, a cluster analysis was performed to examine whether data-driven discrete subgroups of psychoprofiles based on a combination of 6 prominent psychosocial perceptions (ie, trust in the government, beliefs about personal data, social norms, perceived personal and societal benefits, risk perceptions, and self-efficacy) [8,22,23] can be made by grouping users who are associated as much as possible and ensuring that the differences between the groups are as large as possible [21]. By doing this, we obtained insight into a set of characteristics (ie, the psychosocial profile) that describe the groups of (non)users and their behavior on a longitudinal time frame. It is herewith expected that the use of psychosocial profiles, based on psychosocial perceptions, will provide more information about the (non)users of CTAs and their intentions compared to psychosocial characteristics separately.

Based on previous research on psychosocial perceptions related to the coronavirus, it could be expected that people who are concerned about their privacy are less likely to download a CTA [1,9-11,14,16]. However, people who trust the government are more likely to adopt a CTA [9,12,16] and are less likely to have privacy concerns about their personal information being stored or shared [8,22]. In addition, people who have high digital self-efficacy [1,9], a high risk perception of the coronavirus [10,12], or many influences from the social environment (ie, social norms [16]) are assumed to be more likely to download a CTA. Perceived personal and societal benefits also play a role; people who see benefits of using the app will be more likely to download it [9,12,16]. This results in the following research question (RQ) and hypothesis:

RQ1: What clusters or subgroups can be derived from the 6 related psychosocial perceptions about the CM app?

Hypothesis 1 (H1): At least 2 clusters are expected to be identified: (1) A cluster of respondents who have negative perceptions of the CM app. They have low trust in the government, high belief in the use of personal data, and low risk perception and self-efficacy; see few perceived personal and societal benefits of CM; and experience few social norms toward using the CM app. (2) A cluster of respondents who have positive perceptions of the CM app. They have high trust in the government, low belief in the use of personal data, and high risk perception and self-efficacy; see many perceived personal and societal benefits of CM; and experience high social norms toward using the CM app.

If psychosocial perceptions indeed cluster, it is also important to gain more insight into the way they can be characterized. First, the intention to use and adoption associated with clusters are important. In addition, it is of added value to obtain more information about the demographical profile of people in the clusters. Hence, this research aims to examine how these groups can be characterized in terms of demographics, the intention to use the CM app, and CM app adoption.

RQ2: How do the clusters, compiled based on psychosocial perceptions of the CM app, relate to the intention to use the app, the adoption rate of the app, and demographic characteristics?

The clusters distinguished concerning H1 are logically expected to differ significantly in intention to use the CM app and eventual adoption of the CM app. Here, the clusters with predominantly positive perceptions are expected to have a higher intention and adoption rate than the clusters with predominantly negative perceptions.

H2: The cluster(s) with negative perceptions include(s) respondents with a lower intention to use and a lower adoption rate than the cluster(s) with positive perceptions about the CM app.

Additionally, based on the study of Bovens and Wille [24], people who trust the government are expected to be predominantly older as the millennial generation is more critical and skeptical of the performance of political institutions. In addition to increased trust in the government, previous research showed that older people are also expected to have fewer privacy concerns [8,22]. Moreover, older people have a higher risk perception of the COVID-19 virus [14], which is understandable because they have a higher risk to fall seriously ill due to the coronavirus. In contrast, younger people have higher self-efficacy and experience more personal benefits of using a CTA [8,14]. However, based on the results of the uptake of the German [25], Australian [26], and French [27] CTAs, it is expected that the cluster(s) with predominantly positive perceptions of the CM app will consist of relatively older people. The following hypothesis was therefore formulated:

H3: The cluster(s) with negative perceptions about the CM app include(s) younger people than the cluster(s) with positive perceptions.

**Figure 1:**
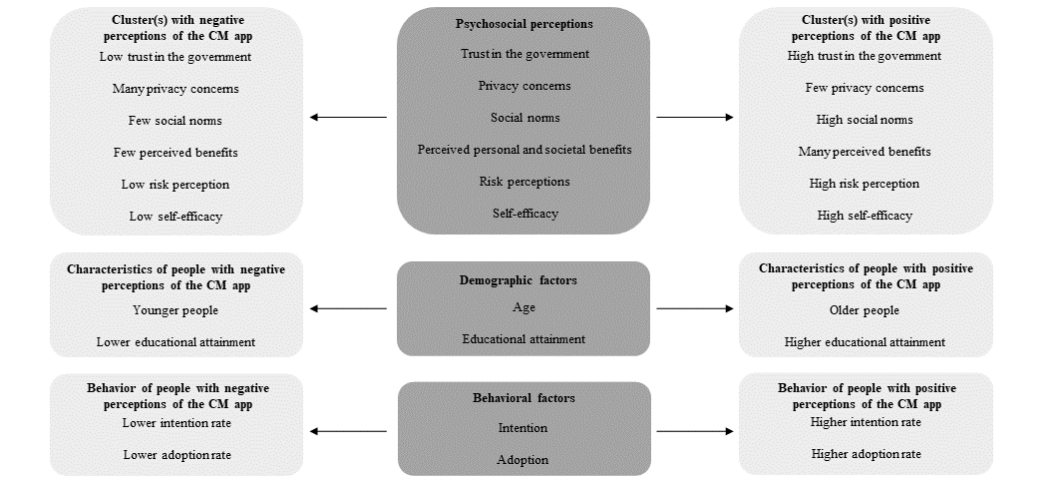
Conceptual framework of the study. CM: CoronaMelder.

In addition to age, the education level has also been found to play a role in expectations regarding the intention to use and the adoption of the CM app [7,8]. With regard to CTAs, people with a lower education level generally have lower self-efficacy; this hinders the adoption of CTAs, because it prevents them from moving on to adoption [1]. People with a lower education level were also found to have fewer privacy concerns [12] and less trust in the government [28]. However, this contradicts the results of Ross [22] and Zetterholm et al [8], who concluded that people with low trust in the government are likely to have more privacy concerns. Moreover, to the best of our knowledge, the relationship between education level and other psychosocial perceptions (ie, risk perceptions, social norms, and perceived personal and societal benefits) has not yet been researched. Therefore, the relationship between clustered psychosocial factors and educational attainment will be explored in this study. Nonetheless, Grill et al [25] found higher adoption of the CM app among those with a higher education level than among respondents with a lower education level. Assuming this, it is expected that in the cluster(s) with a positive perception, there are significantly more respondents with higher educational attainment than in the cluster(s) with a negative perception of the CM app. The hypotheses are conceptualized in Figure 1.

H4: The cluster(s) with negative perceptions about the CM app include(s) respondents with lower educational attainment than the cluster(s) with positive perceptions.

In addition to the psychosocial perceptions believed to determine the intention to use and the adoption of CTAs, the context-related factor health literacy has also been highlighted in the literature to predict the intention to use and the adoption of CTAs. Health literacy refers to “the degree to which individuals have the ability to find, understand, and use information and services to inform health-related decisions and actions for themselves and others” [29]. In the current context, this involves the extent to which a person can understand medical information and fill out medical forms and how often they receive help with this. In the French CTA, the degree of health literacy was found to be predictive of the intention to use and adoption [27]. Therefore, in this study, we investigated whether the context-specific variable health literacy adds to the psychosocial profiles and is therewith predictive of CM-related behavior, in addition to clustering.

RQ3: Does the amount of health literacy predict the intention to use and the adoption of the CM app?

H5: In addition to psychosocial perceptions, the degree of health literacy is predictive of the intention to use and the adoption of the CM app.

## Methods

### Study Design

A longitudinal study was conducted. Data collection took place over 19 months in 6 waves, each of which reflects a different period after the launch of the CM app. This study focused on 2 time points: the baseline measurement (wave 1) and the first follow-up measurement (wave 2) [30]. The data of wave 1 were collected 1.5 weeks after the launch of the CM app, from October 19 to November 1, 2020. At that time, a partial lockdown had been introduced in the Netherlands [31]. Accordingly, the data of wave 2 were collected 1.5 months after the launch of the CM app, from December 7 to 20, 2020. At that point, there was a complete lockdown [31].

### Sampling Procedures

Respondents were recruited from the Longitudinal Internet Studies for the Social Sciences (LISS) panel at Tilburg University. This panel was compiled by Centerdata and the Central Bureau of Statistics (CBS) and consists of around 5000 households across the Netherlands from all strata of the Dutch population [32]. These households vary based on gender, age, ethnicity, income, occupation, and composition. Of the 5000 households, 2093 respondents were selected. The inclusion criteria were being over 16 years of age and having completed both the health and the corona questionnaire of the LISS panel. No use was made of the exclusion criteria.

The longitudinal measurements consisted of a questionnaire that was assessed repetitively within the same sample. Thus, a respondent had to complete the questionnaire of wave 1 to be included in the subsequent measurement of wave 2. Consequently, the final sample consisted of respondents who completed both wave 1 and wave 2 questionnaires.

The questionnaire started with an introduction of the CM app, followed by questions about the respondents’ usage behavior. Based on the indicated behavior (ie, to be or not to be a user of the CM app), routed questions were asked about UTAUT. Thereafter, questions about the respondents’ beliefs about the coronavirus and the CM app, preventive behavior, concepts from the HBM, app-related behavior, and health literacy were asked to both users and nonusers. The questionnaire could be filled in online from any location chosen. To complete the survey, respondents who did not have the necessary equipment (ie, an internet connection or a mobile device) were loaned equipment. LISS panel members received a fee of €7.50 (US $8.04) for completing a survey of 30 minutes. The questionnaire took 7-9 minutes, resulting in a fee of about €2.00 (US $2.14).

### Ethical Considerations

The research data were obtained through the LISS panel [32]. The panelists were assured that their name and address will never be kept along with their responses to ensure privacy. In addition, the participants provided permission to use the data for scientific, social, and policy-relevant research. To process the data, permission was obtained from the Research Ethics and Data Management Committee of the Tilburg School of Humanities and Digital Sciences of Tilburg University (REDC #2020/133a) and the Ethics Committee of the Faculty of Social Sciences, Radboud University (#ECSS-2020-175), Netherlands, by Verpaalen et al [30]. The original informed consent allowed for secondary analysis as the LISS data were released to other researchers after 1.5 years for noncommercial, socially relevant purposes [33]. In addition, a data processing agreement was signed with the commissioner to the LISS panel.

### Variables and Measures

We constructed 6 scales for the 6 psychosocial perceptions (see Multimedia Appendix 1). These scales were standardized using z-scores:

- Trust in the government: The degree of confidence was assessed by the statement “I have confidence in the way the Dutch government is trying to control the coronavirus,” measured on a 7-point Likert scale ranging from 1=“totally disagree” to 7=“totally agree.”
- Beliefs about personal data: The beliefs about personal data were measured using the 2 statements “The CM app keeps track of my location” and “The CM app stores my name or personal data,” measured on a 4-point Likert scale ranging from 1=“definitely not true” to 4=“definitely true”; “I do not know” was coded as a neutral value in the middle of the scale, resulting in a 5-point Likert scale with the choice options “definitely not true,” “maybe not true,” “neutral,” “maybe true,” and “definitely true” (Cronbach *α*=.70). Hence, a higher value on this scale indicates that the respondent has stronger beliefs about the storage of personal data.
- Risk perceptions: The risk perceptions were measured using 6 statements (eg, “I am at risk of infection with the coronavirus in the next 2 months.”). These statements were measured on a 7-point Likert scale ranging from 1=“completely disagree” to 7=“completely agree” (Cronbach *α*=.69).
- Perceived personal and societal benefits: Personal and societal benefits were measured using 6 statements (eg, “The CM app helps to protect people with fragile health from the coronavirus,” “There are personal benefits for me in using the CM app.”). These statements were measured on a 7-point Likert scale ranging from 1=“completely disagree” to 7=“completely agree” (Cronbach *α*=.91).
- Social norms: The degree of social norms was measured using the 2 statements “Many people in my surroundings use the CM app” and “People in my immediate environment think I should use the CM app,” measured on a 7-point Likert scale ranging from 1=“completely disagree” to 7=“completely agree” (Cronbach *α*=.78).
- Self-efficacy: Self-efficacy was measured using 8 statements (e.g., “The CM app is easy to use,” “I have enough technical knowledge to use the CM app”). Two statements were reverse-coded. Hence, all 8 statements were measured on a 7-point Likert scale ranging from 1=“completely disagree” to 7=“completely agree” (Cronbach *α*=.87).

In addition to the 6 psychosocial perceptions, the intention to use the CM app, the adoption of the CM app, and demographics were assessed.

- Intention. The intention was measured using the two statements “I plan to use the CM app in the next 2 months” and “It is likely that I will use the CM app in the next 2 months,” measured on a 7-point Likert scale ranging from 1=“completely disagree” to 7=“completely agree” (Cronbach *α*=.98).
- Adoption. The variable adoption behavior was modified to a dichotomous variable. From 3 categories, 2 categories were created by merging the statements “I have used the CM app in the past, but do not do so currently” and “I have never used the CM app” into 1 category. Hence, adoption was measured on a dichotomous scale with 1=“Yes, I use the CM app” and 2=“No, I do not use the CM app” in order to gain insight into the actual users and nonusers rather than a categorization based on previous behavior.
- Age: Age was included as a continuous variable.
- Educational attainment: The education level of the respondents was categorized as high, medium, or low. According to the CBS [34], primary education and postsecondary vocational education (VMBO) are categorized as lower education; senior general secondary education (HAVO), preuniversity education (VWO), and secondary vocational education (MBO) certificates are for middle education; and higher professional education (HBO) and scientific education (WO) certificates are for higher education.

Finally, the amount of health literacy was measured using 3 questions (eg, “How often is it difficult for you to learn more about your health because you do not fully understand written information?”). Two questions were reversed to match the Likert scale (ie, the higher the score on the Likert scale, the better the health literacy). Accordingly, the 3 statements were combined into 1 scale (Cronbach *α*=.59).

### Statistical Analysis

The data from waves 1 and 2 were imported into SPSS Statistics version 28 (IBM Corp.) and merged into 1 file. Consequently, to test what clusters or subgroups could be derived, agglomerative hierarchical cluster analysis was performed on the data of wave 1. We examined whether the psychosocial perceptions of respondents regarding the CM app were related and formed clusters (all measured in wave 1). The Ward minimum variance clustering technique was used with the squared Euclidean distance as the metric. Based on clustering, an agglomeration schedule was set up as well as a dendrogram (see Multimedia Appendix 2). Moreover, inverse scree plots of the Ward total within-group sums of squared errors of successive cluster solutions were constructed and compared to determine the optimal number of clusters.

Thereafter, we analyzed how the different clusters could be defined in terms of the intention to use and the adoption of the CM app and demographic data in wave 1. To test the differences between the clusters in the intention to use the CM app and the adoption of the CM app, 1-way ANOVA and cross-tabulation analysis with the chi-square test were performed. Consequently, we tested whether the clusters differed significantly in age using another 1-way ANOVA. Moreover, cross-tabulation analysis and the chi-square test were performed to examine whether there were significant differences between the clusters in respondents’ educational attainment.

Lastly, we investigated whether there were variables that were predictive of the intention to use or the adoption of the CM app in wave 2. First, linear regressions were performed to measure the influence on the adoption of the CM app. The linear regressions consisted of 5 sequentially added blocks: clusters, demographics (ie, educational attainment, age, gender), health literacy, adoption in wave 1, and significant interaction terms thereof (ie, health literacy × age). All independent variables were measured in wave 1. With these regressions, we measured whether these variables predicted the intention to use the CM app. Afterward, logistic regressions were performed measuring the influence of clusters, demographics (ie, education level, age, gender), health literacy, intention and adoption in wave 1, and significant interaction terms thereof (ie, adoption in wave 1 × educational attainment) to predict the adoption of the CM app using the same blocks. Again, all independent variables were measured in wave 1.

## Results

### Respondents’ Characteristics

In wave 1, 2093 respondents were invited to participate in the survey. Of these respondents, 8.7% (183/2093) did not respond to the invite and 0.5% (10/2093) did not complete the survey in its totality. This resulted in a total of 1900 (90.8%) respondents in wave 1. Of these, 27.2% (517/1900) did use the CM app and 71.2% (1352/1900) did not.

In wave 2, 1895 people were invited, of which 15.1% (287/1895) did not reply to the invite and 0.7% (14/1895) did not complete the survey. This resulted in a total of 1594 (84.1%) respondents in wave 2. Of these, 31.3% (499/1594) did use the CM app and 68.7% (1095/1594) did not. An overview of the respondents’ demographics, intention to use, and adoption in waves 1 and 2 is provided in Table 1.

**Table 1:** Overview of the demographics of waves 1 and 2.

Characteristics and categories			Wave 1 respondents (N=1900)		Wave 2 respondents (N=1594)
**Gender, n (%)**					
	Female	1045 (55.0)		866 (54.3)	
	Male	855 (45.0)		728 (45.7)	
**Age (years), mean (SD)**					
	17-96	51.8 (18.3)		53.3 (18.1)	
**Education level, n (%)**					
	Low	431 (22.7)		426 (26.8)	
	Middle	658 (34.7)		552 (34.7)	
	High	740 (39.0)		612 (38.4)	
	Other	71 (3.7)		0	
Intention to use, mean (SD)			3.9 (2.1)		3.6 (2.2)
**Adoption, n (%)**					
	Yes, I use the CM^a^ app.	517 (27.2)		499 (31.3)	
	No, I do not use the CM app.	1383 (72.8)		1095 (68.7)	

^a^CM: CoronaMelder.

### Cluster Analysis

**Figure 2:**
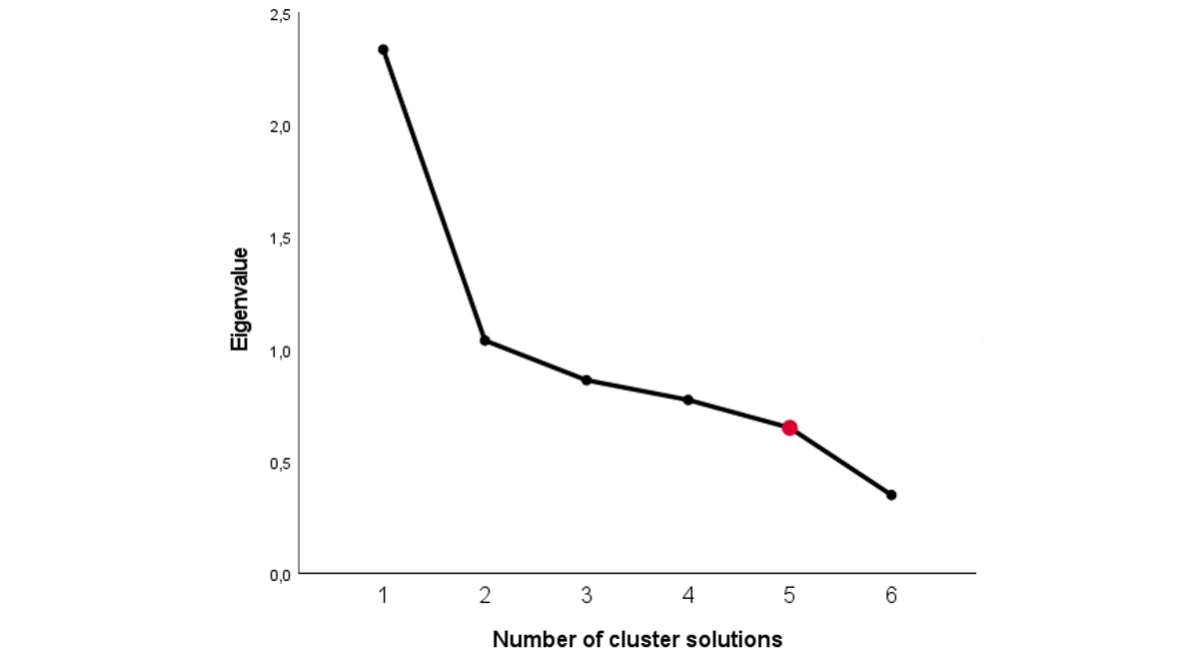
Scree plot.

Hierarchical cluster analysis was performed on the data of wave 1. Here, the solutions with 1-6 clusters were examined and compared using a scree plot (see Figure 2), a dendrogram (see Multimedia Appendix 2), and frequency tables (see Multimedia Appendix 3).

Eventually, a 5-cluster solution was chosen because based on the frequency tables. This solution contained the most equal distribution of respondents, with 275 (14.5%) respondents in the smallest cluster and 500 (26.3%) respondents in the largest cluster. Based on the dendrogram, this 5-cluster solution also seemed a stable clustering solution.

### Psychosocial Perceptions in Clustered Subgroups

**Figure 3:**
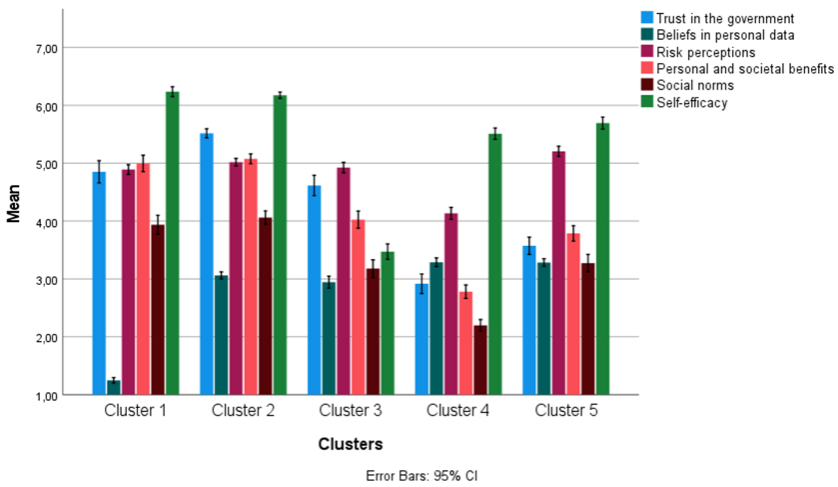
Distribution of the psychosocial perceptions across the 5 clusters.

Figure 3 shows how these clusters differed from each other in terms of the interrelated 6 psychosocial perceptions. Moreover, Table 2 shows the mean scores for the scales of the 6 psychosocial perceptions per cluster. From both the figure and the table, we could derive that cluster 1 was a cluster with above-average trust in the government, perceived personal and social benefits, social norms, self-efficacy, and risk perceptions. The beliefs about personal data in cluster 1 were well below the average. Cluster 2 matched this, except that respondents in the cluster had higher beliefs about personal data than the average. Cluster 4 also had above-average beliefs about personal data, but the other psychosocial perceptions were lower than the average. Cluster 3 was a mostly neutral cluster, with all psychosocial perceptions centered around the mean, except for self-efficacy. Finally, cluster 5 was a diverse cluster, with both negative and positive perceptions. For example, people in cluster 5 generally had below-average trust in the government and perceived benefits but above-average risk perceptions, beliefs about personal data, social norms, and self-efficacy.

**Table 2:** Test statistics of the 6 psychosocial perceptions by cluster for wave 1 (N=1900).

Psychosocial perceptions	Cluster 1 (n=275), mean (SD)	Cluster 2 (n=500), mean (SD)	Cluster 3 (n=349), mean (SD)	Cluster 4 (n=435), mean (SD)	Cluster 5 (n=341), mean (SD)	Overall mean (SD)	*F*_4,1895_(*χ*^2^), *P* value
Trust in the government	4.77 (1.61)	5.53 (0.80)	4.62 (1.34)	2.94 (1.62)	3.54 (1.21)	4.31 (1.66)	268.021, <.001
Risk perceptions	4.91 (0.68)	4.98 (0.69)	4.84 (0.71)	4.10 (0.99)	5.23 (0.74)	4.81 (0.86)	121.093, <.001
Personal and societal benefits	4.97 (1.19)	5.08 (0.87)	3.97 (1.10)	2.74 (1.13)	3.87 (1.07)	4.14 (1.39)	336.359, <.001
Social norms	3.92 (1.36)	4.04 (1.18)	3.18 (1.13)	2.19 (0.99)	3.34 (1.21)	3.33 (1.37)	169.743, <.001
Self-efficacy	6.23 (0.69)	6.18 (0.56)	3.40 (1.06)	5.50 (0.93)	5.63 (0.87)	5.54 (1.23)	689.038, <.001
Beliefs about personal data	1.32 (0.47)	3.71 (0.85)	3.51 (0.97)	3.99 (0.97)	4.02 (0.74)	2.83 (0.96)	533.273, <.001

### Demographic and Behavioral Differences Between Clustered Subgroups

To test whether there were significant differences between the clusters in the intention to use the CM app, 1-way ANOVA was performed on wave 1 data. As can be seen in Table 3, ANOVA results showed a significant difference between the clusters in the intention value (*F*_4,1899_=275.1, *P*<.001). Bonferroni analysis revealed that the intention to use the CM app was significantly higher in cluster 1 than in cluster 3 (mean difference between 2 clusters, M_dif_=2.306, 95% CI 1.93-2.68, *P*_adjusted_<.001), cluster 4 (M_dif_*=*3.103, 95% CI 2.74-3.46, *P*_adjusted_ <.001), and cluster 5 (M_dif_*=*1.680, 95% CI 1.30-2.06, *P*_adjusted_<.001). Likewise, the intention to use the CM app in cluster 2 was significantly higher than in cluster 3 (M_dif_=2.287, 95% CI 1.96-2.61, *P*_adjusted_ <.001) and cluster 4 (M_dif_=3.083, 95% CI 2.78-3.39, *P*_adjusted_<.001). The intention to use the CM app was the lowest in cluster 4 and differed significantly from cluster 3 (M_dif_=0.797, 95% CI 0.46-1.13, *P*_adjusted_ <.001) and cluster 5 (M_dif_=–1.423, 95% CI –1.76 to –1.08, *P*_adjusted_<.001). Lastly, the intention to use the CM app in cluster 3 was significantly lower than that in cluster 5 (M_dif_=–0.626, 95% CI –0.98 to –0.27, *P*_adjusted_<.001). A bar chart of the comparison of clusters based on the intention to use the CM app can be found in Multimedia Appendix 4, Figure D3.

**Table 3:** Demographics, intention to use, and adoption by cluster for wave 1.

Characteristics			Cluster 1 (n=275)		Cluster 2 (n=500)		Cluster 3 (n=349)		Cluster 4 (n=435)		Cluster 5 (n=341)	Overall mean (SD)		*F*(*χ*^2^), *P* value	
Age (years), mean (SD)			53.52 (16.94^a,b,c^)		49.04 (17.57^a,d^)		63.95 (16.51^b,d,e,f^)		46.21 (17.27^c,e^)		49.27 (17.58^f^)	51.8 (18.3)		*F*_4,1895_=60.6, <.001	
Intention to use, mean (SD)			5.36 (1.85^a^)		5.34 (1.66^a,b,c^)		3.05 (1.62)		2.25 (1.44)		3.68 (1.85^b,c^)	3.92 (2.10)		*F*_4,1895_=275.1, <.001	
**Adoption, n (%)**															*F*_4,1900_=527.6, <.001
	Yes	169 (61.5)		255 (51.0)		20 (5.7)		16 (3.7)		57 (16.7)		N/A^g^	N/A		
	No	106 (38.5)		245 (49.0)		329 (94.3)		419 (96.3)		284 (83.3)		N/A	N/A		
**Education level, n (%)**															*F*_8,1896_=152.4, <.001
	Low	43 (15.6)		87 (17.4)		169 (48.7)		117 (26.9)		79 (23.3)		N/A	N/A		
	Middle	87 (31.6)		175 (35.0)		103 (29.7)		164 (37.7)		132 (38.9)		N/A	N/A		
	High	145 (52.7)		238 (47.6)		75 (21.6)		154 (35.4)		128 (37.8)		N/A	N/A		

^a-f^Means in the same row that do not share superscripts differ at *P*=.05. Hence, corresponding superscripts indicate that the values differ significantly from each other.

^g^N/A: not applicable.

Next, a chi-square test of association was performed to test whether there was an association between the clusters and the adoption of the CM app in wave 1. There appeared to be a significant association (*χ*^2^_4=_527.568, *P*<.001). As shown in Table 3 and Multimedia Appendix 4 (Figure D4), clusters 3, 4, and 5 did have predominantly nonadopters, cluster 2 had as many adopters as nonadopters, and cluster 1 had predominantly adopters.

Accordingly, 1-way ANOVA was performed to test whether there were significant differences between clusters by age. On average, people were 51.82 years old (SD 18.27). ANOVA results showed a significant difference between clusters by age (*F*_4,1895_=60.586, *P*<.001). Bonferroni analysis revealed that respondents in cluster 1 were significantly older than in cluster 2 (M_dif_*=*4.480, 95% CI 0.85-8.11, *P*_adjusted_ =.005). However, the respondents in cluster 3 were significantly older than in cluster 1 (M_dif_*=–*10.426, 95% CI –14.33 to –6.52, *P*_adjusted_<.001), cluster 2 (M_dif_*=–*14.906, 95% CI –18.28 to –11.53, *P*_adjusted_<.001), cluster 4 (M_dif_*=*17.734, 95% CI 14.26-21.21, *P*_adjusted_<.001), and cluster 5 (M_dif_*=*14.676, 95% CI 10.99-18.36, *P*_adjusted_<.001). Cluster 4 was the youngest cluster and differed significantly from cluster 1 (M_dif_*=*7.309, 95% CI 3.58-11,04, *P*_adjusted_<.001). This is also visualized in the bar chart in Multimedia Appendix 4, Figure D1.

Lastly, a chi-square test of association was performed to test the association between clusters and the respondents’ educational attainment. There was a significant association (*χ*^2^_8_=152.372, *P*<.001). Cluster 3 comprised respondents with the lowest education level, clusters 4 and 5 comprised respondents with a middle education level, and clusters 1 and 2 comprised respondents with the highest education level (see Multimedia Appendix 4, Figure D2).

### Predictors of the Intention to Use and the Adoption of the CM App

With regard to the prediction of the respondents’ intention to use and adoption of the CM app in wave 2, multiple regression analyses were performed. The influential factors for the intention to use the CM app were measured using linear regression (see Table 4), while the influential factors for the effective adoption of the CM app were measured using logistic regression (see Table 5).

**Table 4:** Linear regression analysis to longitudinally explain the intention to use the CM^a^ app in wave 2.

Model			Step 1 (R^2^=0.527)							Step 2 (R^2^=0.528)							Step 3 (R^2^=0.535)							Step 4 (R^2^=0.726)				
		B (SE)		*t* (*df*)		*P* value		B (SE)			*t* (*df*)		*P* value		B (SE)			*t* (*df*)		*P* value		B (SE)			*t* (*df*)		*P* value	
**Model 1**																												
	Cluster^b^ 2	–0.019 (0.125)		–0.154 (4,1899)		.877		–0.019 (0.126)			–0.154 (4,1899)		.878		–0.028 (0.126)			–0.218 (4,1899)		.827		–0.231 (0.102)			2.265 (4,1899)		.024	
	Cluster 3	–2.306 (0.135)		–17.125 (4,1899)		<.001		–2.306 (0.141)			–16.396 (4,1899)		<.001		–2.357 (0.131)			–16.414 (4,1899)		<.001		–0.818 (0.125)			–6.529 (4,1899)		<.001	
	Cluster 4	–3.107 (0.129)		–24.146 (4,1899)		<.001		–3.109 (0.131)			–23.747 (4,1899)		<.001		–3.110 (0.166)			–23.772 (4,1899)		<.001		–1.626 (0.115)			–14.121 (4,1899)		<.001	
	Cluster 5	–1.679 (0.135)		–12.403 (4,1899)		<.001		–1.679 (0.137)			–12.266 (4,1899)		<.001		–1.697 (0.137)			–12.368 (4,1899)		<.001		–0.532 (0.116)			–4.572 (4,1899)		<.001	
**Model 2**																												
	Middle^b^ education	N/A^c^		N/A		N/A		–0.023 (0.103)			–0.222 (8,1899)		.824		0.003 (0.104)			0.027 (8,1899)		.979		0.012 (0.083)			0.148 (8,1899)		.883	
	High education	N/A		N/A		N/A		–0.009 (0.102)			–0.090 (8,1899)		.929		0.030 (0.104)			0.289 (8,1899)		.773		0.007 (0.084)			0.078 (8,1899)		.938	
Age			N/A		N/A		N/A		–0.005 (0.042)			–0.117 (8,1899)		.907		0.005 (0.042)			1.129 (8,1899)		.897		–0.077 (0.034)			–2.271 (8,1899)		.023
Gender			N/A		N/A		N/A		–0.006 (0.078)			–0.076 (8,1899)		.939		0.006 (0.078)			0.082 (8,1899)		.935		0.011 (0.063)			0.172 (8,1899)		.864
**Model 3**																												
	Health literacy	N/A		N/A		N/A		N/A			N/A		N/A		–0.071 (0.041)			–1.738 (9, 1899)		.082		–0.023 (0.033)			–0.681 (9, 1899)		.496	
**Model 4**																												
	Adoption in wave 1	N/A		N/A		N/A		N/A			N/A		N/A		N/A			N/A		N/A		–2.623 (0.082)			–32.014 (10, 1899)		<.001	

^a^CM: CoronaMelder.

^b^The variables “cluster 1” and “low education” were constants.

^c^N/A: not applicable.

**Table 5:** Logistic regression analysis to longitudinally explain the adoption of the CM^a^ app in wave 2.

Model		Step 1 (R^2^=0.328)				Step 2 (R^2^=0.333)				Step 3 (R^2^=0.334)				Step 4 (R^2^=0.727)		
		Exp(B)	95% CI	*P* value	Exp(B)		95% CI	*P* value	Exp(B)		95% CI	*P* value	Exp(B)		95% CI	*P* value
**Model 1^b^**																
	Cluster 2	0.631	0.452-0.882	.007	0.649		0.462-0.911	.012	0.641		0.456-0.900	.032	0.661		0.377-1.160	.148
	Cluster 3	0.051	0.031-0.082	<.001	0.047		0.029-0.079	<.001	0.045		0.027-0.075	<.001	0.440		0.210-0.919	.029
	Cluster 4	0.043	0.027-0.070	<.001	0.046		0.028-0.075	<.001	0.046		0.028-0.074	<.001	0.569		0.271-1.193	.135
	Cluster 5	0.162	0.110-0.239	<.001	0.167		0.113-0.247	<.001	0.163		0.110-0.242	<.001	0.702		0.372-1.324	.275
**Model 2^c^**																
	Middle education	N/A^d^	N/A	N/A	1.133		0.806-1.594	.472	1.177		0.832-1.665	.522	1.495		0.903-2.477	.118
	High education	N/A	N/A	N/A	1.250		0.895-1,746	.190	1.319		0.934-1.863	.302	1.429		0.860-2.375	.168
	Age	N/A	N/A	N/A	1.171		1.023-1.341	.022	1.185		1.034-1.359	.023	1.104		0.899-1.357	.345
	Gender^e^	N/A	N/A	N/A	1.211		0.943-1.556	.134	1.227		0.954-1.578	.106	1.416		0.968-2.073	.073
**Model 3**																
	Health literacy	N/A	N/A	N/A	N/A		N/A	N/A	0.872		0.704-1.081	.058	1.033		0.752-1.419	.843
**Model 4**																
	Intention to use in wave 1	N/A	N/A	N/A	N/A		N/A	N/A	N/A		N/A	N/A	0.046		0.029-0.073	<.001
	Adoption in wave 1^f^	N/A	N/A	N/A	N/A		N/A	N/A	N/A		N/A	N/A	1.791		1.553-2.064	<.001

^a^CM: CoronaMelder.

^b^All clusters were dummy-coded, with cluster 1 as the reference category.

^c^Educational attainment was dummy coded, with low education as reference category.

^d^N/A: not applicable.

^e^Men were coded as 0 and women as 1.

^f^“Yes, I use the CM app” (coded 0) or “No, I do not use the CM app” coded 1).

#### Intention to Use

In model 1, the cluster variable (including the 4 dummy variables relative to cluster 1 that was coded as a reference category) was a significant predictor of the intention to use the CM app in wave 2 for clusters 3, 4, and 5. Cluster 2 was not significant relative to the reference category (ie, cluster 1). In model 2, the demographic variables educational attainment, age, and gender, as well as the amount of health literacy in model 3, did not appear to be significant predictors of the intention to use the CM app. However, in model 4, the adoption of the CM app in wave 1 was a significant predictor of the intention to use the app in wave 2. The higher the adoption in wave 1, the lower the intention to use in wave 2. Lastly, the health literacy × age interaction did not significantly predict the intention to use the CM app (*P*=.149).

#### Adoption

In model 1, all 4 dummy cluster variables (with cluster 1 as the reference category) appeared to be significant predictors of the adoption of the CM app in wave 2. Hence, with a β between 0 and 1, clusters 2, 3, 4, and 5 tended to have lower odds of moving to adoption than cluster 1 (see Tables 3 and 5). In model 2, age did significantly predict adoption as well; adoption increased with increasing age. Model 3 showed that health literacy was not a significant predictor of the adoption of the CM app, although the *P* value was close to significance (*P*=.058). Higher health literacy insignificantly predicted lower adoption. Next, based on model 4, the intention to use and the adoption of the CM app in wave 1 were significant predictors of adoption in wave 2. A higher intention to use the app in wave 1 predicted higher adoption in wave 2, whereas higher adoption in wave 1 predicted lower adoption in wave 2. Finally, the interaction of the adoption of the CM app in wave 1 and educational attainment was not a significant predictor of adoption, again with a *P* value close to significance (*P*=.060).

## Discussion

### Principal Findings

To answer RQ1 (whether subgroups or clusters could be derived based on psychosocial perceptions), cluster analysis was performed and 5 clusters were inspected accordingly. H1 was confirmed; at least 2 clusters could be distinguished, of which 1 cluster had predominantly positive perceptions of the CM app (ie, cluster 1) and 1 cluster had predominantly negative perceptions (ie, cluster 4). These clusters could therefore be labeled the “pro–CM app group” and the “contra–CM app group,” respectively. Cluster 3 was mostly neutral, while cluster 5 had both positive and negative perceptions. These could be labeled the “neutral CM app group” and the “mixed-attitude CM app group,” respectively. Finally, cluster 2 was mostly positive but did have more concerns about personal data, so it could be labeled the “pro-but-privacy-cautious CM app group.”

The clusters were predictive of the adoption of the CM app. Overall, Table 5 showed that clusters 2-5 had a lower probability of proceeding to adoption than the reference cluster 1. Moreover, the findings revealed that there was a clear difference between the clusters with regard to demographics, the intention to use the CM app, and the adoption of the CM app. The respondents in the pro–CM app group (cluster 1) were significantly older and had a higher education level than those in the contra–CM app group (cluster 4). This finding confirms H3 and H4 (ie, the cluster with negative perceptions about the CM app includes younger people than the cluster with positive perceptions, and the cluster with negative perceptions about the CM app includes respondents with lower educational attainment than the cluster with positive perceptions). Moreover, as expected, the intention to use and the adoption of the CM app were higher in the positive clusters than in the negative clusters. Therefore, H2 (the cluster with negative perceptions about the CM app includes respondents with a lower intention and adoption rate than the cluster with positive perceptions) could also be accepted. In doing so, however, we found that the relatively positive cluster 2 was not a significant predictor of the intention to use the CM app compared to cluster 1 as a reference category. This might be explained by the fact that cluster 2 is an average-to-positive group with regard to perceptions about the CM app, not characterized by a specific psychosocial perception. In contrast, participants in the other clusters scored divergent on at least 1 psychosocial perception, which may explain why cluster 2 had less predictive power compared to the other clusters. Furthermore, all other clusters turned out to have specific demographic characteristics. For example, cluster 3 was characterized by a relatively high age, whereas cluster 1 contained mostly highly educated people. As such, the clustering revealed interesting insights into the psychosocial profiles in relationship with the intention to use, behavior, and demographic characteristics.

Moreover, there were a few other unexpected results. The clusters were predictive of the intention to use and the adoption of the CM app in wave 2, which is in line with papers that have put forward these psychosocial perceptions as being explanatory of the adoption of CTAs [5,13-16]. However, when the intention to use and the adoption of the CM app from wave 1 were added to the model, the clusters had less predictive power. Thus, the intention to use and the adoption of the CM app in wave 1 had a greater predictive value than the clusters. This means that the clusters are predictive to a certain degree, but above all, they are distinctive for adopters and nonadopters.

Furthermore, the ratio between these adopters and nonadopters was not quite as expected based on their clustering profiles. In cluster 2, psychosocial perceptions were mostly positive, except for the beliefs about personal data, which is why we also expected that there would be mostly adopters within this cluster. However, there were about as many adopters (51.0%) as nonadopters (49.0%). The same was the case for cluster 3, in which all psychosocial perceptions were around the average, except self-efficacy. This cluster hardly contained any adopters (5.7%). When comparing the clustering profiles in Figure 3, we can see that the profiles of, for example, clusters 1 and 2 are aligned but differ only on the beliefs about personal data. This could indicate that the anomalous items are either decisive for not adopting the CM app or the factor that causes a divergent adoption rate between the clusters.

Additionally, there seems to be a discrepancy between the intention to use and the adoption of the CM app. In the data of wave 1, it was notable that the intention to use the CM app was almost equal and relatively high in clusters 1 and 2, but the adoption of the CM app in wave 2 prevailed only in cluster 1 (65.5%). In addition, while the intention to use the CM app in cluster 5 was fairly average (ie, 3.68 vs 3.92), there were only 23.4% of respondents in that cluster who downloaded the CM app in wave 2. Because the results showed a positive relationship between the intention to use the CM app in wave 1 and the adoption of the CM app in wave 2, this discrepancy is difficult to explain. However, people who showed high adoption of the CM app in wave 1 had a low intention to use the app in wave 2. This suggests that people may have had negative experiences using the CM app in the period from wave 1 to wave 2, which made them decide to stop using the app. In addition, people might not have had an active experience with the CM app. When the CM app is installed on a device, the app works immediately. No further actions need to be taken in the app, and little feedback is provided by the app, which could also give people the feeling that the app is not operative. In this regard, it would also be of added value to investigate the correlation between users’ expectations and experiences accordingly or whether something else is causing the intention and adoption rates to decline over time with CTAs in general.

There is, for example, an age difference between the clusters related to self-efficacy. Age was not a significant predictor of the intention to use the CM app but was one for the adoption of the CM app. In line with Horstmann et al [9] and Thorneloe et al [1], this could imply that self-efficacy is an important barrier to adopting a CTA. According to Van Gemert-Pijnen et al [21], especially the elderly and those with lesser language or digital skills may experience difficulty in adopting a CTA. It might, therefore, be the case that younger people with relatively higher self-efficacy and skills download the app immediately, while older people move to adoption later with, for example, the help of relatives or do not adopt it at all. This also explains why a higher age predicted lower adoption for wave 1, whereas it was the other way around for wave 2: the lower adoption of the CM app by older people in wave 1 might be explained by (the lack of) self-efficacy, which forms a barrier. Younger people, however, predominantly adopted the app in wave 1 and might have had a negative user experience, as discussed earlier, resulting in lower adoption of the CM app in wave 2.

Another explanation could be the nuance of the variable “beliefs in personal data.” This variable refers to the belief that the CM app keeps track of the users’ location and personal data, with a higher value indicating stronger beliefs about the storage of personal data. Although these statements do not contain a value judgment and the fact that a stronger belief does not necessarily lead to privacy concerns, the literature shows that there are many privacy concerns among CTA users [1,9-11,14,16]. This might explain why hardly any adoption took place in cluster 2, while all psychosocial perceptions were positive, except for the beliefs about personal data. Even more so, it might explain the decline in intention and adoption rates over time. The CM app requires active permission from the user to establish a (working) Bluetooth connection in order for it to work. If the Bluetooth connection is not active, the app signals the user that it is not working. For people with high privacy concerns, this might fuel privacy concerns, causing them to stop using the CM app.

### Theoretical and Practical Implications

This study has some theoretical and practical implications. The results showed that the psychosocial clustering profiles were predictive of the intention to use the CM app and the adoption of the CM app. With this, insight was gained into CM app adopters and the way future intention and adoption rates could be predicted. According to Clatworthy [35], this can help determine which groups might best benefit from interventions. In the case of the CM app, the clustering profiles can be used to target campaigns or promotional materials to people with specific clustering profiles.

We recommend focusing promotional activities mainly on clusters 2, 3, and 5. Cluster 1, the pro–CM app group, was in favor of the CM app, and reinforcement is therefore not necessary. Cluster 2, the pro-but-privacy-cautious CM app group, was mainly positive but experiences privacy concerns. Therefore, within promotional activities, it is important to pay sufficient attention to the privacy of CM app users, the anonymity of data, and the data retention policy. Cluster 3, the neutral CM app group, had perceived benefits and social norms just below the average and low self-efficacy. For people with this set of psychosocial perceptions, it is important to emphasize personal and societal benefits. Additionally, through promotional activities, an attempt should be made to increase their self-efficacy, for example, by explaining how the app works or by emphasizing the user-friendliness of the app. Next, with cluster 5, the mixed-attitude CM app group, the trust in the government was well below the average. Meanwhile, beliefs about personal data were well above the average. Hence, information should be carefully compiled, showing that the app created by the government can be trusted and that data are handled carefully and anonymously. Finally, cluster 4 was the contra–CM app group with low trust in the government, low risk perception, low perceived benefits and social norms, and high beliefs in personal data. Here, the focus should be on the risks of being infected with COVID-19. By increasing this perception of risk, the perceived benefits could be increased, as well as the perceived need to adopt the app among this group of people. However, this group is predominantly negative, and the likelihood of this group moving to adoption is relatively low. Therefore, we recommend focusing on this cluster to a lesser extent when setting up promotional activities.

In addition to clustering profiles, age can be considered. For example, it is expected that younger people will be less likely to adopt the CM app than older people. Thus, it is of added value to target any campaign or communication strategy regarding a CTA to people of lower age.

### Limitations and Future Research

This study shows the importance of approaching psychosocial perceptions in cluster form. Targeting specific clustering profiles could ultimately increase the adoption of a CTA. It should, however, be noted that a CTA is not always comparable to an eHealth app in a broader sense. For example, a CTA is mostly developed by the government, whereas an eHealth app is predominantly developed by a health institution or provider. In addition, a CTA is not actively used, whereas an eHealth app often has several functionalities with which the user engages. Hence, with a CTA, adoption is installing the app, whereas with eHealth, there can be a distinction between app adoption and use. Finally, a CTA is meant for adoption by an entire population, whereas an eHealth app also often focuses on a particular target group (eg, patients with chronic obstructive pulmonary disease [COPD] or cancer, smokers). It would therefore be interesting to investigate in follow-up research whether intention and adoption can be predicted using the psychosocial profiles for other eHealth apps.

Additionally, the generalizability of these results is subject to certain limitations. For instance, as stated by Clatworthy et al [35], there is a prominent need for guidelines for conducting and reporting cluster analyses within health psychology. There is currently no absolute and verifiable method for the validation of clusters within cluster analysis. Therefore, no validation could be performed in this study. However, in this study, useful and visually distinguishable clusters were identified. The statistical difference between clusters was confirmed by the statistical tests performed in this study.

Moreover, a methodological limitation concerns the reliability of the variables measuring health literacy. Health literacy had a Cronbach *α* of .59, which is considered poor. The Cronbach *α* could not be increased by removing 1 of the 3 items that made up the scale. The health literacy scale should therefore be interpreted with caution.

In addition, the intention scale consists of the statements “I plan to use the CM app in the next 2 months” and “I plan to continue using the CM app in the next 2 months.” These statements measure the intention of both users and nonusers to use the CM app in the subsequent 2 months. Here, the starting point for users is adherence, while for nonusers, it refers to the intention to start using the app. According to the literature, these behaviors may have other underlying reasons (eg, expectations for the intention to use vs user experience for adherence). This could have had an effect on the findings, but given a CTA where there is no active use of the app, this effect is estimated to be negligible.

Lastly, we recommend improving inclusiveness concerning the methodology of this study. Respondents who did not have an internet connection or a mobile device were provided with the supplies to complete the survey, such as a laptop. However, no support or guidance was provided in completing the survey. People who have, for example, low self-efficacy or a low education level might be less likely to participate in a study that has to be conducted individually on a computer. With that, people who had a low education level or lesser digital skills were likely to be underrepresented in this study. Thus, in a follow-up study, attention should be paid to the representativeness of the sample by offering support in completing the questionnaire.

### Conclusion

The beliefs in different domains on the CM app were clustered (eg, trust in the government, self-efficacy), and these clustering profiles were predictive of the intention to use and behavior. This study provides insight into the profiles of CM (non)intenders and (non)adopters.

This study also contributes to the literature with more information about additional determinants, such as health literacy, that cause users to intend to use the CM app and eventually adopt the app. In line with UTAUT and the HBM, this revealed that clustering profiles are important and of added value to determine the intention to use a CTA and the adoption of a CTA. These insights could be applied to the development of successive CTAs to improve their inclusiveness and accessibility, for example, by targeting campaigns to people with a particular psychosocial profile.

## Appendix

Multimedia Appendix 1 Standardized scales of the (psychosocial) variables and corresponding Cronbach *α*.

Multimedia Appendix 2 Dendrogram.

Multimedia Appendix 3 Frequency tables.

Multimedia Appendix 4 Bar charts comparing the clusters.

## Abbreviations

CBS: Central Bureau of Statistics
CM: CoronaMelder
CTA: contact-tracing app
HBM: Health Belief Model
LISS: Longitudinal Internet Studies for the Social Sciences
RQ: research question
UTAUT: unified theory of acceptance and use of technology

## Data Availability

The original data file can be retrieved through the Longitudinal Internet Studies for the Social Sciences panel. The minimalized data file used in this study can be obtained from the corresponding author upon reasonable request and used for noncommercial purposes only.
