# Supplementary material for: The Adoption of a COVID-19 Contact-Tracing App: Cluster Analysis
Source: JMIR Form Res. 2023 Jun 20;7:e41479. doi: 10.2196/41479 (PMC10284059; doi:10.2196/41479)
Supplement: Multimedia Appendix 1 [file formative_v7i1e41479_app1.docx]

## Appendix A – Standardized Scales of the (Psychosocial) Variables and Corresponding Cronbach Alpha

| Trust in the government  α x | 1. I have confidence in the way the Dutch government is trying to control the coronavirus |
| --- | --- |
| Beliefs about personal data  α .697 | 1. The CoronaMelder app keeps track of my location 2. The CoronaMelder app stores my name or personal data |
| Risk perceptions  α .687 | 1. I am at risk of infection with the coronavirus in the next two months 2. There is a high probability that I will become infected with the coronavirus in the next two months 3. If I become infected with the coronavirus, there is a good chance that I will infect others 4. I am concerned about becoming infected with the coronavirus 5. Being infected with the coronavirus has major physical, psychological or economic consequences for me 6. I mind if I infect other people with the coronavirus |
| Perceived personal and societal benefits  α .907 | 1. By using the CoronaMelder app, I am helping to combat the coronavirus 2. The CoronaMelder app is helpful in making sure the coronavirus is spreading less 3. Using the CoronaMelder app helps the Dutch economy 4. Using the CoronaMelder makes you a good citizen 5. The CoronaMelder app helps to protect people with fragile health from the coronavirus 6. There are personal benefits for me in using the CoronaMelder app |
| Social norms  α .782 | 1. Many people in my surroundings use the CoronaMelder app 2. People in my immediate environment think I should use the CoronaMelder app |
| Self-efficacy  α .866 | 1. It costs me a lot of time and energy to use the CoronaMelder app 2. The CoronaMelder app is easy to use 3. I think it costs me a lot of time and energy to use the CoronaMelder app 4. I think the CoronaMelder app is easy to use 5. I have enough (technical) knowledge to use the CoronaMelder app 6. I am able to use the CoronaMelder app 7. I know how to install apps on my phone 8. I know how to turn Bluetooth on and off on my phone |

| Intention  α .975 | 1. I plan to use the CoronaMelder app in the next 2 months 2. It is likely that I will use the CoronaMelder app in the next 2 months |
| --- | --- |
| Adoption | 1. Yes, I use the CM app 2. No, I do not use the CM app |
| Health literacy  α .585 | 1. How often does someone help you read letters or leaflets from your GP, the hospital or other health care institutions? 2. How often is it difficult for you to learn more about your health because you do not fully understand written information? 3. How confident are you that you are filling out medical forms correctly yourself? |
