# Supplementary figures and images for: The Adoption of a COVID-19 Contact-Tracing App: Cluster Analysis
Source: JMIR Form Res. 2023 Jun 20;7:e41479. doi: 10.2196/41479 (PMC10284059; doi:10.2196/41479)

## Appendix B - Dendrogram


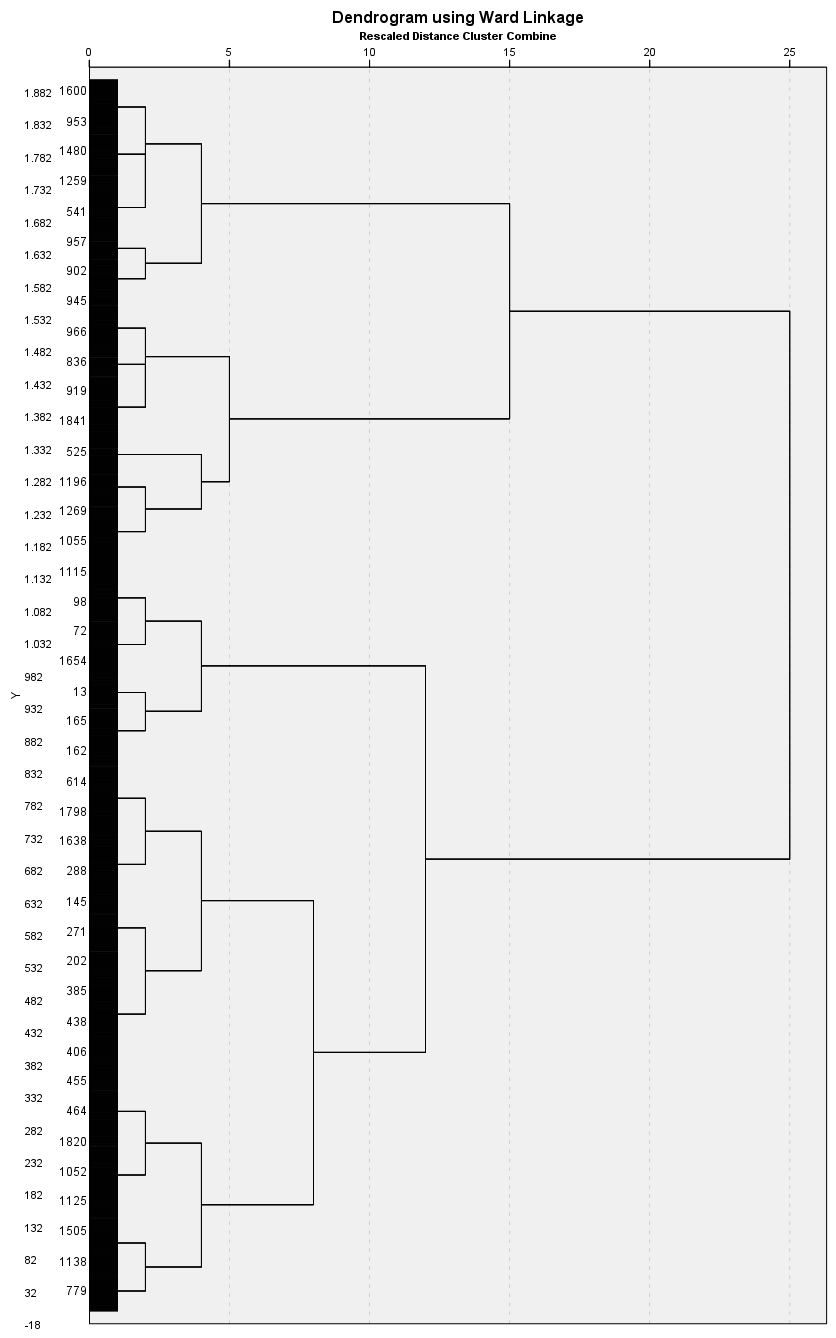

Supplement: Multimedia Appendix 2 [file formative_v7i1e41479_app2.docx]
