# Supplementary material for: The Adoption of a COVID-19 Contact-Tracing App: Cluster Analysis
Source: JMIR Form Res. 2023 Jun 20;7:e41479. doi: 10.2196/41479 (PMC10284059; doi:10.2196/41479)
Supplement: Multimedia Appendix 3 [file formative_v7i1e41479_app3.docx]

## Appendix C – Frequency tables

2 cluster solution

|  | Frequency | Percent | Cumulative percent |
| --- | --- | --- | --- |
| Cluster 1 | 1116 | 58,7 | 58,7 |
| Cluster 2 | 784 | 41,3 | 100 |

3 cluster solution

|  | Frequency | Percent | Cumulative percent |
| --- | --- | --- | --- |
| Cluster 1 | 1116 | 58,7 | 58,7 |
| Cluster 2 | 349 | 18,4 | 77,1 |
| Cluster 3 | 435 | 22,9 | 100 |

4 cluster solution

|  | Frequency | Percent | Cumulative percent |
| --- | --- | --- | --- |
| Cluster 1 | 275 | 14,5 | 14,5 |
| Cluster 2 | 841 | 44,3 | 58,7 |
| Cluster 3 | 349 | 18,4 | 77,1 |
| Cluster 4 | 435 | 22,9 | 100 |

5 cluster solution

|  | Frequency | Percent | Cumulative percent |
| --- | --- | --- | --- |
| Cluster 1 | 275 | 14,5 | 14,5 |
| Cluster 2 | 500 | 26,3 | 40,8 |
| Cluster 3 | 349 | 18,4 | 59,2 |
| Cluster 4 | 435 | 22,9 | 82,1 |
| Cluster 5 | 341 | 17,9 | 100 |

6 cluster solution

|  | Frequency | Percent | Cumulative percent |
| --- | --- | --- | --- |
| Cluster 1 | 275 | 14,5 | 14,5 |
| Cluster 2 | 500 | 26,3 | 40,8 |
| Cluster 3 | 349 | 18,4 | 59,2 |
| Cluster 4 | 220 | 11,6 | 70,7 |
| Cluster 5 | 341 | 17,9 | 88,7 |
| Cluster 6 | 215 | 11,3 | 100 |
