# Supplementary material for: The Adoption of a COVID-19 Contact-Tracing App: Cluster Analysis
Source: JMIR Form Res. 2023 Jun 20;7:e41479. doi: 10.2196/41479 (PMC10284059; doi:10.2196/41479)

## Appendix D – Bar charts comparing the clusters

Figure D1. Comparison of clusters by age.


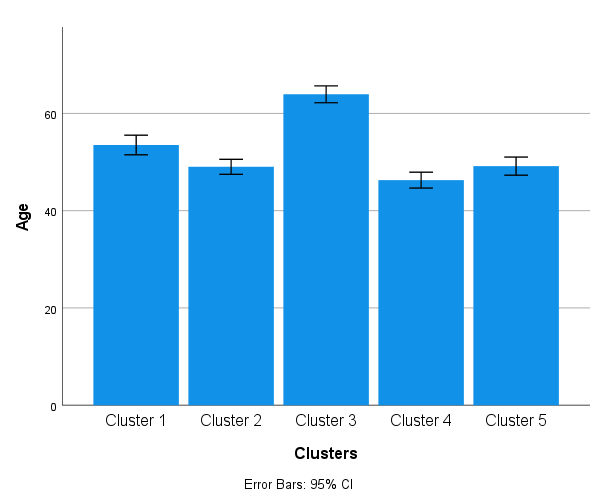


Figure D2. Comparison of clusters by educational attainment.


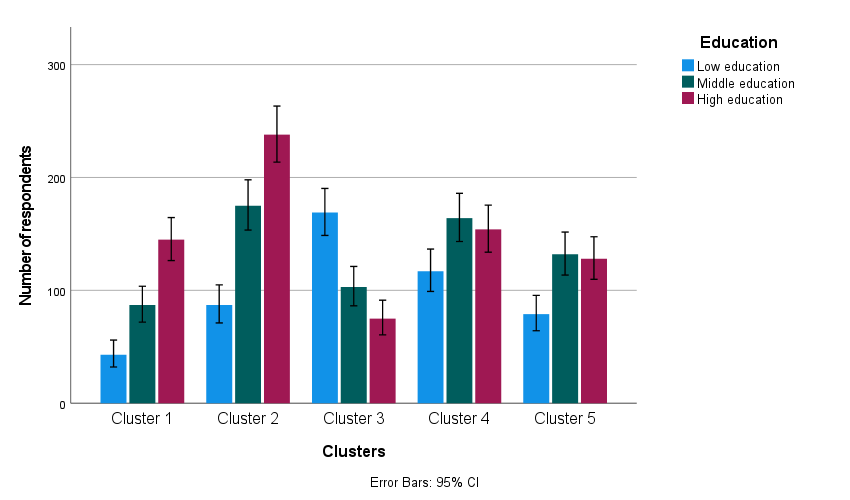


Figure D3. Comparison of clusters by intention.


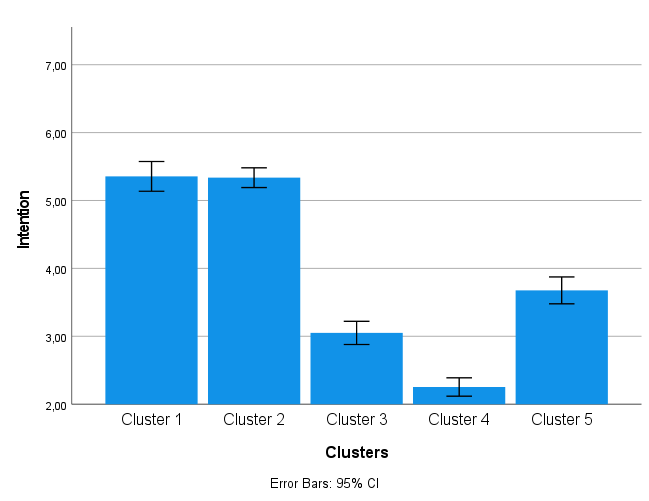


Figure D4. Comparison of clusters by adoption.


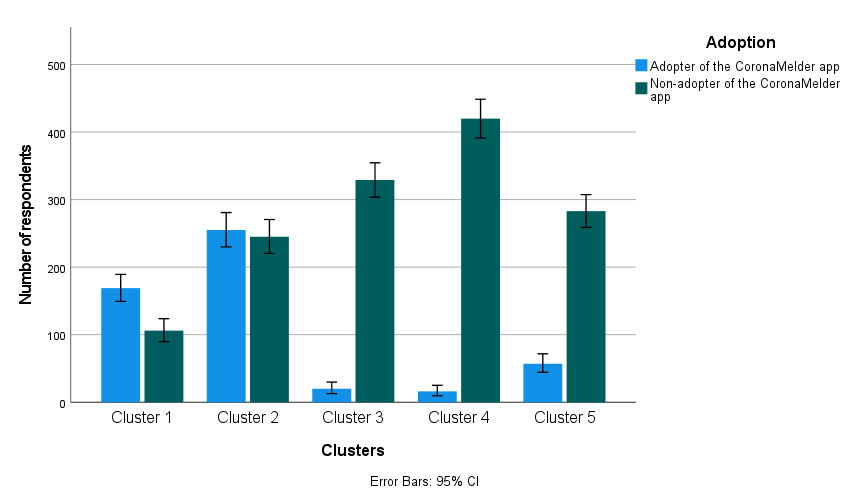

Supplement: Multimedia Appendix 4 [file formative_v7i1e41479_app4.docx]
